# Maternal consumption of artificially sweetened beverages during pregnancy is associated with infant gut microbiota and metabolic modifications and increased infant body mass index

**DOI:** 10.1080/19490976.2020.1857513

**Published:** 2020-12-31

**Authors:** Isabelle Laforest-Lapointe, Allan B. Becker, Piushkumar J. Mandhane, Stuart E. Turvey, Theo J. Moraes, Malcolm R. Sears, Padmaja Subbarao, Laura K. Sycuro, Meghan B. Azad, Marie-Claire Arrieta

**Affiliations:** a Physiology and Pharmacology, University of Calgary, Calgary, Alberta, Canada; b Pediatrics, University of Calgary, Calgary, Alberta, Canada; c Biologie, Université De Sherbrooke, Sherbrooke, Québec, Canada; d Alberta Children’s Hospital Research Institute (ACHRI), Calgary, Alberta, Canada; e Snyder Institute for Chronic Diseases, University of Calgary, Calgary, Alberta, Canada; f Pediatrics and Child Health, University of Manitoba, Winnipeg, Manitoba, Canada; g Pediatrics, University of Alberta, Edmonton, Alberta, Canada; h British Columbia Children’s Hospital, The University of British Columbia, Vancouver, British Columbia, Canada; i Hospital for Sick Children, Toronto, Ontario, Canada; j Pediatrics, University of Toronto, Toronto, Ontario, Canada; k Medicine, McMaster University, Hamilton, Ontario, Canada; l Microbiology, Immunology, and Infectious Diseases, University of Calgary, Calgary, Alberta, Canada

**Keywords:** Gut microbiota, 16s rRNA, metabolomics, artificial sweeteners, genomics, obesity, gestational exposure, breastfeeding, BMI, Child cohort

## Abstract

Artificial sweetener consumption by pregnant women has been associated with an increased risk of infant obesity, but the underlying mechanisms are unknown. We aimed to determine if maternal consumption of artificially sweetened beverages (ASB) during pregnancy is associated with modifications of infant gut bacterial community composition and function during the first year of life, and whether these alterations are linked with infant body mass index (BMI) at one year of age. We studied 100 infants from the prospective Canadian CHILD Cohort Study, selected based on maternal ASB consumption during pregnancy (50 non-consumers and 50 daily consumers). BMI was higher among ASB-exposed infants. Infant stool (16S rRNA gene sequencing) and urine (untargeted metabolomics) were acquired in early (3–4 months) and late (12 months) infancy. We identified four microbiome clusters, of which two recapitulated the maturation trajectory of the infant gut bacterial communities from immature (Cluster 1) to mature (Cluster 4) and two deviated from this trajectory (Clusters 2 and 3). Maternal ASB consumption did not differ between clusters, but was associated with community-level shifts in infant gut bacterial taxonomy structure and depletion of several *Bacteroides* sp. in Cluster 2. In the complete dataset, urine succinate and spermidine levels at 3 months were higher in ASB-exposed infants, and urine succinate was positively associated with BMI at one-year-old. Overall, gestational exposure to ASB was associated with gut microbiota structure in infants from Cluster 2, and gut microbiota structure was associated with infant BMI. Gestational exposure to ASB was positively associated with infant urine succinate and spermidine. Succinate was found to mediate 29% of the effect of ASB exposure on BMI at one-year-old, revealing a potential role of this metabolite in increased infant weight linked to gestational ASB consumption. As we face an unprecedented rise in childhood obesity, future studies should evaluate the causal relationships between maternal ASB consumption (a modifiable exposure), gut microbiota and metabolites, infant metabolism, and body composition.

## Introduction

Childhood obesity in the United States increased from 5% to 18.5% between 1978 and 2016,^1^ magnifying the risk of cardiometabolic disease and mental health disorders later in life.^2^ Recent work from the CHILD Cohort Study showed that maternal consumption of artificially sweetened beverages (ASB) during pregnancy is associated with higher infant body mass index (BMI) at one year of age.^3^ Importantly, this association was independent of key obesity risk factors, such as maternal BMI, smoking, poor diet, diabetes, short breastfeeding duration, and earlier introduction of solid food.^3^ Similar associations have been reported in several other prospective birth cohorts,^4^ but the underlying mechanism has not been studied.

The gastrointestinal tract, a key site for host metabolic regulation,^5,6^ is colonized by a vast community of microbes including bacteria, viruses, and micro-eukaryotes.^7^ The gut microbiome is highly heterogeneous during infancy, characterized by colonization patterns^8–10^ that are influenced by the maternal microbiome,^11,12^ method of birth,^13–15^ infant nutrition (breast milk or formula),^16–18^ and antibiotic treatment.^14,19^ Simultaneously, important aspects of metabolic development occur during this period of life, many of which rely on interactions between microbes and host cells.^20^ Recent studies in mice show that artificial sweetener consumption during pregnancy predisposes offspring to increased weight gain through behavioral (i.e. preference for sweet foods, appetite increase) and physiological mechanisms (i.e. stimulation of intestinal sugar absorption, increased postnatal weight gain, altered lipid profiles, downregulation of hepatic detoxification, and increased insulin resistance).^21–24^

Common low-calorie sweeteners include synthetic artificial sweeteners (e.g. non-acesulfame-potassium, aspartame, advantame, neotame), sugar alcohols (e.g. erythritol, xylitol), and plant-based sweeteners (e.g. sucralose, thaumatin, monk fruit).^25^ The effects of artificial sweeteners on the gut microbiome are diverse, including impacts on composition and function (see Suez *et al*.^26^ for a synthesis). Suez *et al*.^27^ also demonstrated that artificial sweetener consumption in adult mice directly impacts gut microbiome composition and function, leading to an increase in host glucose intolerance. More recently, Stichelen *et al*.^24^ addressed gestational exposure to artificial sweeteners, finding changes in bacterial metabolites and a decrease in *Akkermansia municiphila* in the pups’ gut microbiome. However, the consequences of maternal artificial sweetener consumption during pregnancy on the infant gut microbiota have not been reported in humans.

To address this knowledge gap and build on our prior observations in the CHILD Cohort Study, we evaluated the association of maternal artificially sweetened beverage consumption during pregnancy with the infant gut microbiota in a subset of 100 infants (50 with daily maternal ASB consumption during pregnancy and 50 unexposed controls; see Table 1 for maternal participants’ characteristics). We employed next-generation sequencing of the 16S rRNA amplicon gene combined with a community typing analysis (Dirichlet Multinomial Mixtures [DMM] modeling)^28^ and urine untargeted metabolomics to understand if ASB intake was associated with a shift in infant microbiota composition and function that might explain the relationship between maternal ASB intake during pregnancy and infant BMI at one year of age.

**Table 1.** Participant characteristics for mothers exposed or unexposed to ASB_s_ during pregnancy

**Participant** **Characteristics**	Unexposed* to ASB			Exposed* to ASB			
	**[N = 50]**			**[N = 50]**			
**Other variables**	**mean**	**(SD)**	**(range)**	**mean**	**(SD)**	**(range)**	**p**
**Added Sugars (g)**	58.3	(29.4)	(20.0–152.7)	68.3	(47.8)	(19.7–342.3)	0.21
**Breastfeeding (months)**	8.0	(7.1)	(0.0–24.0)	7.6	(7.4)	(0.0–24.0)	0.68
**Education (years)**	16.6	(2.6)	(12.0–24.0)	16.4	(2.5)	(11.0–22.0)	0.80
**Gestational Age (weeks)**	39.2	(1.4)	(35.0–42.0)	38.9	(1.4)	(35.0–41.0)	0.38
**Gestational Weight Gain**	30.8	(12.5)	(5.0–65.0)	37.3	(35.3)	(5.0–224.0)	0.29
**Healthy Eating Index-2010 (score)**	72.6	(9.1)	(55.2–86.1)	71.6	(7.1)	(57.7–91.2)	0.54
**Maternal Age (years)**	33.2	(4.28)	(23.7–42.8)	32.6	(4.6)	(20.5–40.9)	0.36
**Maternal Body Mass Index**	26.0	(6.0)	(17.6–40.5)	27.5	(6.1)	(19.3–42.1)	0.24
**Total Sugar (g)**	141.6	(54.2)	(47.0–255.9)	141.0	(80.4)	(49.5–586.0)	0.96
**Matching variables**	**N**	**n**	**%**	**n**		**%**	**p**
**Sex** *Female* *Male*	46 54	23 27	46.0 54.0	23 27		46.0 54.0	1.00
**Birth mode** *Vaginal* *C-section*	64 36	32 18	64.0 36.0	32 18		64.0 36.0	1.00
**Breastfeeding (3 months)** *Exclusive* *Partial* *None*	32 30 38	16 15 19	32.0 30.0 38.0	16 15 19		32.0 30.0 38.0	1.00
**Breastfeeding (12 months)** *No* *Yes* *Missing*	68 28 4	34 14 2	68.0 28.0 4.0	34 14 2		68.0 28.0 4.0	1.00
Child Antibiotics (oral/IV) 3–12 M** No *Yes* *Missing*	54 20 26	27 10 13	54.0 20.0 26.0	27 10 13		54.0 20.0 26.0	1.00
**Maternal Body Mass Index** *Normal* *Overweight* *Missing*	46 51 3	26 23 1	52.0 46.0 2.0	20 28 2		40.0 56.0 4.0	0.45
**Ethnicity** *Asian* *Caucasian* *First Nations* *Other*	10 81 5 4	7 37 3 3	14.0 74.0 6.0 6.0	3 44 2 1		6.0 88.0 4.0 2.0	0.33

*Unexposed = no consumption by the mother during pregnancy; Exposed = daily consumption

**Antibiotics before 3 months is an exclusion criterion

## Results

### Microbiome clusters

**Figure 1.**
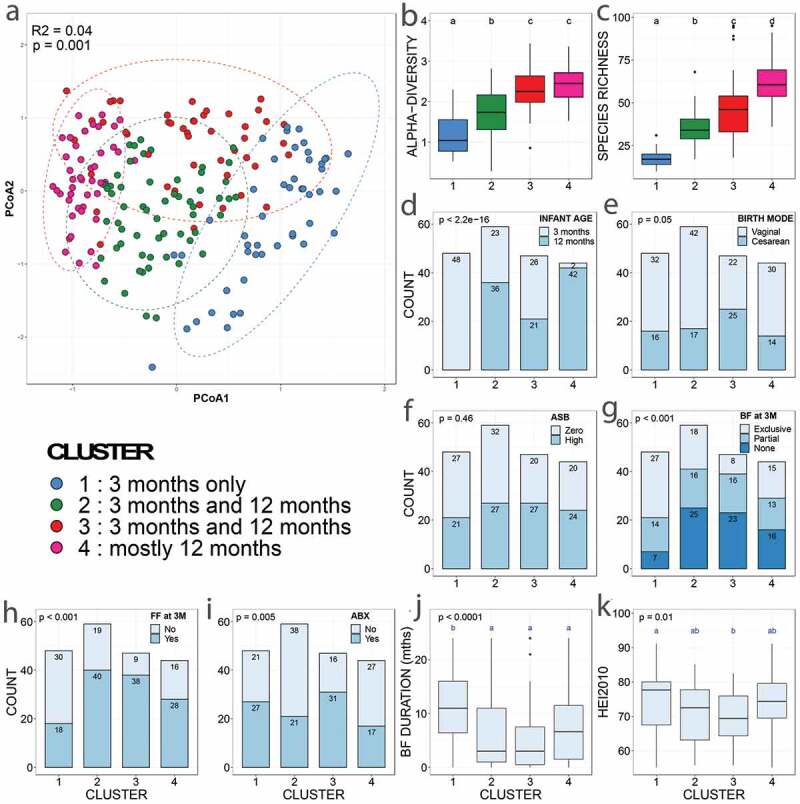
Discrepancies in covariate distribution, alpha- and beta-diversity between microbiota clusters

**Figure 2.**
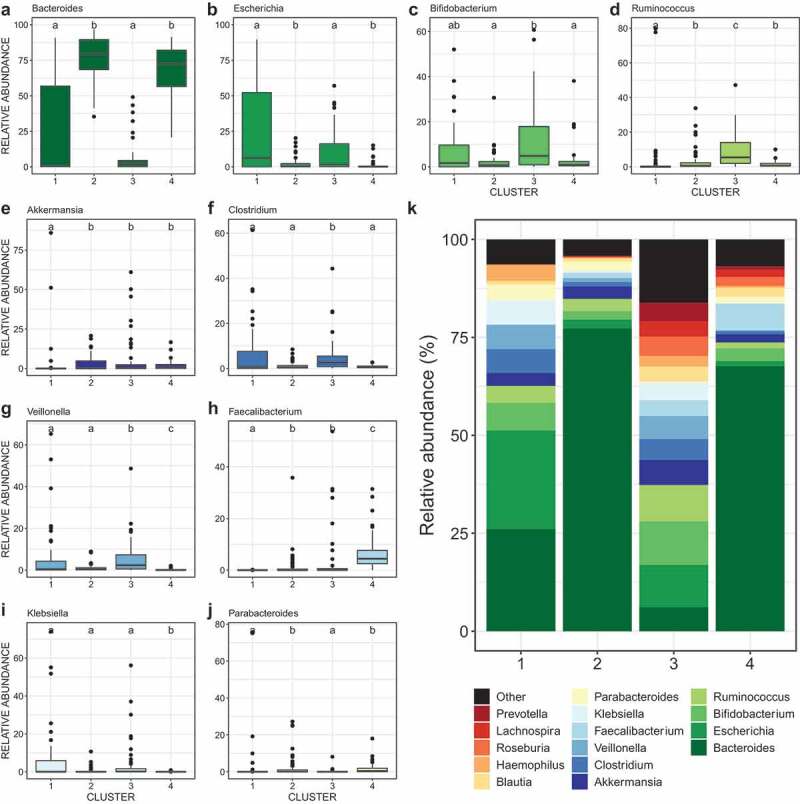
Differences in relative abundances of the dominant bacterial genera between clusters

We performed community typing analysis based on Dirichlet Multinomial Mixtures (DMM) modeling^28^ to identify clusters of similar bacterial community structure amongst our samples. Based on their microbiota composition, the infant fecal samples clustered in four groups (Figure 1–2 and eFigure 1). Gut bacterial species richness (Figure 1b), alpha- (Figure 1c) and beta-diversity (Figure 1a) and taxonomic composition (Figure 2) differed between clusters, reflecting broad community differences. Clusters 1 and 4 comprised microbial communities reflecting the well-described effect of temporal maturation during the first year of life; with cluster 1 comprising only three-month (3 M) samples and cluster 4 comprising almost exclusively twelve-month (12 M) samples. Clusters 2 and 3 comprised a mixture of 3 M and 12 M samples. Compared to the other three clusters, cluster 1 showed a higher proportion of exclusive breastfeeding. Cluster 3 included a higher proportion of mothers receiving intrapartum antibiotics, infants born by C-section, and formula feeding (Figure 1). However, there was no difference in maternal ASB consumption between clusters, suggesting that this exposure did not influence the compositional differences that drove cluster classification (Figure 1f). In addition, the clusters did not differ in terms of maternal sugar intake, gestational diabetes, age, ethnicity, education, maternal gestational antibiotics, study site, infant antibiotics, or infant or mother secretor status.

(A) Principal component analysis (PCoA) ordinations of variation in beta-diversity of infant gut bacterial communities based on Bray-Curtis dissimilarities among samples. Ellipses represent 95% confidence intervals. (B-C) Box plots showing the alpha-diversity (richness and Shannon’s diversity) per DMM cluster. The central line denotes the median, the boxes cover the 25th and 75th percentiles, and the whiskers extend to the most extreme data point, which is no more than 1.5 times the length of the box away from the box. Points outside the whiskers represent outlier samples. Letters denoted significant differences (non-parametric Kruskal-Wallis test followed by post-hoc test of Dunn with FDR correction following Benjamini-Hochberg method; *P* < .05). (D-K) Variable distribution between clusters tested with non-parametric Kruskal-Wallis test followed by either a post-hoc generalized linear model (glm) with a binomial/logistic distribution (D-I) or (J-K) a post-hoc Dunn test with FDR correction following Benjamini-Hochberg method. Minuscule letters indicate statistical differences between clusters from post-hoc generalized linear model (glm) with a binomial/logistic distribution. “BF at 3 M” stands for “breastfeeding at three months” and “FF at 3 M” for “formula feeding at three months”. Aside from maternal ASB consumption (F), only the variables that showed a statistical difference in distribution between clusters are presented. No differences were found for maternal age, ethnicity, education, diabetes; study site, household pets, siblings, or introduction of solid foods at 3 or 6 months. Cluster 1 included 48 samples from 48 infants; cluster 2 included 59 samples from 49 infants; cluster 3 included 47 samples from 39 infants; and cluster 4 included 44 samples from 43 infants. See methods for definition of variables. For all clusters with the same letter, the difference between groups is not statistically significant. If two variables have different letters, they are significantly different.

(A-J) Relative abundance across DMM clusters of the ten most dominant bacterial genera and (K) of the 15 most dominant bacterial genera. Letters indicate significant differences between clusters (non-parametric Kruskal-Wallis test, post-hoc Dunn test with Benjamini-Hochberg FDR correction). For all clusters with the same letter, the difference between groups is not statistically significant. If two variables have different letters, they are significantly different. Cluster 1 contains only three months of age. Clusters 2 and 3 are composed of a mix three and twelve months of age, and Cluster 4 only 12 M (except two samples).

### Relative association of ASB on microbial community structure

**Figure 3.**
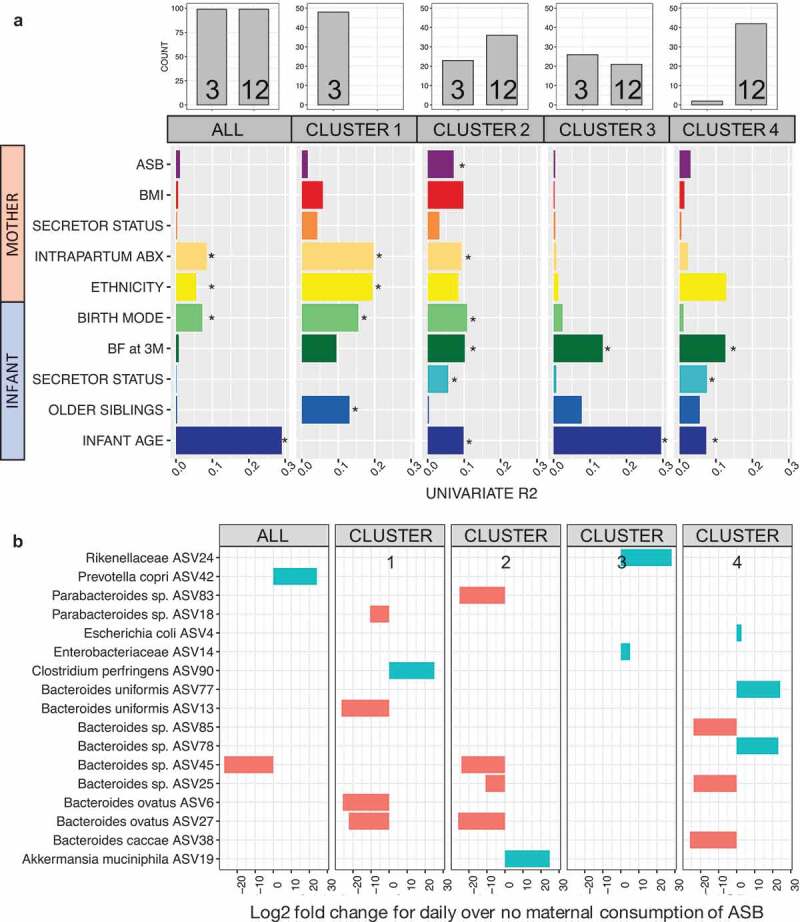
Drivers of gut bacterial beta-diversity and indicator taxa associated with maternal consumption of ASB differ between clusters

*Envfit* analysis (univariable models) identified thirteen variables as significant drivers of gut bacterial beta-diversity from which we selected eight non-redundant variables to build our models: infant age, maternal intrapartum antibiotics, maternal ethnicity, birth mode, breastfeeding status at three months, presence of older siblings, infant secretor status, and maternal ASB consumption (Figure 3a and eFigure 2). Considering the complete dataset, the significant predictors were infant age, maternal ethnicity, intrapartum antibiotics, and birth mode. The same four variables, plus breastfeeding status at 3 months, were tested in a PERMANOVA (multivariable model), altogether explaining 14.2% of community variance (Table 2). Maternal ASB consumption was a significant predictor of infant gut bacterial composition only in the multivariable model (R^2^ = 0.7%; Table 2). Birth mode (vaginal vs. C-section), intrapartum antibiotics, and breastfeeding status at three months had also a significant influence on community composition (respectively R^2^ = 0.8%, 1.7%, and 1.9%), but to a lesser extent than infant age (R^2^ = 7.3%) and mother’s ethnicity (R^2^ = 2.5%; Table 2).

Univariate models showing significance and explained variance of 10 variables on bacterial community structure across all data and each cluster subset. Horizontal bars show the amount of variance (R^2^) explained by each covariate in the model as determined by *envfit*. Asterisk denotes the significant covariates in each data subset (*P* < .05). All 32 variables considered in this study are shown in eFigure 2. In this figure, ASB represents artificially sweetened beverages and BF at 3 M represents infant’s breastfeeding status at three months (see methodology). (B) 14 bacterial taxa identified as significant features associated with maternal consumption of ASB by DESeq2.

**Table 2.** Maternal consumption of ASB during pregnancy is associated with bacterial community assembly during the first year of life. Permutational Analysis of Variance (PERMANOVA) of gut bacterial community composition (Bray-Curtis dissimilarities) testing associations with different explanatory variables. The model on the complete dataset (ALL) accounts for repeated measures. The set of variables to be tested was chosen based on results from univariate *envfit* models

**Variables**	**All** (R^2^%)	**Cluster 1** (R^2^%)	**Cluster 2** (R^2^%)	**Cluster 3** (R^2^%)	**Cluster 4** (R^2^%)
Infant age (3 M vs. 12 M)	7.3***	8.5*	4.1***	8.0***	3.9**
Ethnicity	2.5***	NS	NS	NS	NS
Breastfeeding at 3 M	1.9***	5.1**	5.0***	6.0*	6.4**
Maternal Intrapartum Abx	1.7***	NS	NS	NS	NS
Birth mode	0.8**	NS	NS	NS	NS
Older siblings	NS	NS	NS	NS	NS
Infant secretor status	NS	NS	NS	NS	NS
Maternal ASB	0.7*	NS	3.2**	NS	NS
**Total R^2^ (%)**	**15.1**	**13.6**	**9.1**	**14.0**	**10.3**

^NS^*P* > 0.05, * *P* < 0.05, ** *P* < 0.01, *** *P* < 0.001

Next, we repeated the beta-diversity analyses separately within each of the four clusters. *Envfit* univariable models identified distinct drivers for each cluster (Figure 3a). Interestingly, the drivers of beta-diversity in cluster 1 (only 3 M samples) were mainly maternal factors (i.e. birth mode, mother’s ethnicity, intrapartum antibiotics) whereas the drivers of cluster 4 (mostly 12 M) were infant factors (infant’s secretor status, breastfeeding at three months, and infant age (Figure 3a). Cluster 2 was the only cluster in which maternal ASB consumption was associated with beta-diversity (R^2^ = 3.2%), and this association was confirmed by the univariable (Figure 3a, eFigure 2) and multivariable (Table 2) analyses.

We tested for associations of specific bacterial features in the infant gut with maternal ASB consumption. In the complete dataset, we identified two ASVs associated with maternal consumption of ASB, one species being depleted (*Bacteroides* sp. ASV45, log2 fold change = −27.2 and another species enriched (*Prevotella copri* ASV42, 24.2) among infants exposed to high maternal ASB intake (Figure 3b). Repeating this test within each cluster, we identified 15 additional ASVs enriched or depleted. For cluster 2, one ASV was enriched (ASV19, *Akkermansia municiphila*, 24.9) and four depleted (*Bacteroides ovatus* ASV27, −25.9; Parabacteroides sp. ASV83, −25.2; *Bacteroides* sp. ASV45, −24.9; *Bacteroides* sp. ASV25, −10.7) with maternal ASB consumption (Figure 3b). All adjusted *p*-values were below 0.001 and corrected with Benjamini-Hochberg for FDR.

### Association between ASB exposure and urine metabolites

**Figure 4.**
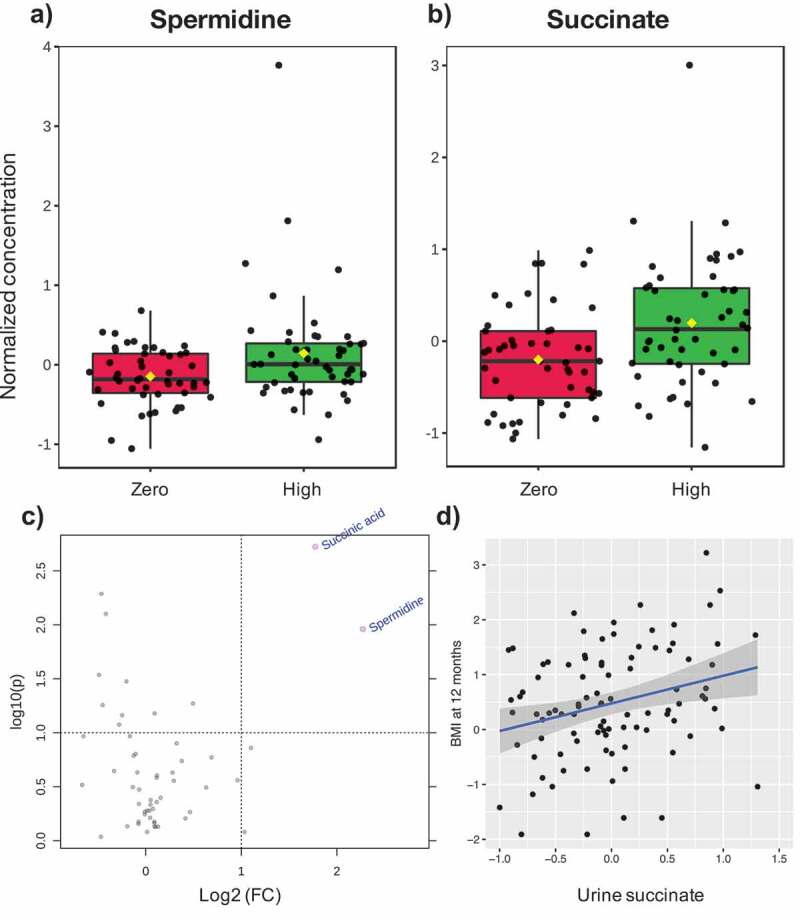
Association between maternal ASB consumption, urine metabolite concentration in 3 M-old infants, and infant BMI at 12 months

Using the software MetaboAnalyst,^29^ we identified twenty metabolites that varied across clusters (eFigure 3). Since functional features in the metabolome are reflective of microbial metabolism redundancy, and less susceptible to interindividual variability typical of taxonomic datasets, we tested for a significant effect of maternal ASB consumption on the complete dataset at 3M and 12M old. Two urine metabolites, spermidine (log2 fold change = 2.27, *p* = .01) and succinate (log2 fold change = 1.77, *p* = .001), were significantly higher in 3M old infants exposed to ASB (Figure 4).

Significant changes were detected in the normalized concentrations of (A) spermidine (log2 fold change = 2.27, *p* = .01) and (B) succinate (log2 fold change = 1.77, *p* = .001) in infant urine metabolomics by (C) volcano plot (combination of fold change and t-tests). (D) Linear regression of BMI at 12 months against normalized succinate concentration. Metabolite concentrations were normalized by median, log transformed and transformed with pareto scaling (mean-centered and divided by the square root of the standard deviation of each variable) with the software MetaboAnalyst.

### Association of ASB, gut microbiome and urine metabolites with infant BMI at one-year-old

Finally, using a multivariable linear model on the complete dataset, we tested the association of maternal ASB consumption, microbial community composition and the two metabolites linked to high ASB consumption with infant BMI z-score at one year of age. In this cohort, infant birth weight was not correlated with BMI at one-year-old (*p* = .28). However, our multivariable linear model confirmed that daily maternal ASB consumption is associated with higher infant BMI (ß-estimate = 0.42, 95%CI 0.03:0.80, *P* = .037; Table 3), and showed that BMI was associated with the microbiome composition at 12 months (PCoA1 axis; ß-estimate = −0.71, 95%CI −1.40:-0.01, *P* = .048; Table 3) but not at three months (not shown). These results suggest that features of PCoA1 (i.e. lower relative abundance of *Bacteroidetes* and *Faecalibacterium*, and higher relative abundance of *Escherichia, Klebsiella, Bifidobacterium, Haemophilus, Clostridium*, and *Veillonella*; eFigure 4) are inversely associated with infant BMI. Notably, succinate was positively associated with BMI (ß-estimate = 0.45, 95%CI 0.15:0.76, *P* = .004; Table 3, Figure 4d) but not spermidine (*P* = .49), revealing that this association may also involve microbial-derived metabolites. We then performed a mediation analysis which showed that urine succinate mediated 29% of the observed association between ASB and BMI at one-year-old (mediation effect of ß-estimate = 0.29, 95%CI 0.03:2.19, *P* = .04).

**Table 3.** Maternal consumption of ASB during pregnancy and urine succinate are associated with higher infant BMI at one-year-old. Linear models showing the explanatory power of maternal ASB consumption and urine succinate at 3 M old on infant BMI z-score at one year old, as well as the two main axes of ordination of bacterial community structure (beta-diversity) on samples acquired at 3 M and 12 M old. The full models are: [1]BMI at 1y ~ Succinate; [2]BMI at 1y ~ ASB + PCoA1 + PCoA2. Microbial variables were transformed (squared root and order quantile normalized respectively) to achieve normality. Here we present only the best model for 12 months fitted by stepwise selection by Akaike information criterion because we detected no association between BMI at one year old and microbiota composition at 3 M old

**Variables**	**Infant BMI z-score at 1 year**				**Total adj. R^2^**
	ß-est.	95% CI	P-value	R^2^	
*Model 1* Urine succinate (3 months)	**0.45**	**[0.15,0.76]**	**0.004**	**7.4%**	**7.4%**
*Model 2* Maternal ASB (daily vs. no consumption)	**0.42**	**[0.03,0.81]**	**0.037**	**4.1%**	**8.1%**
PCoA axis 1	**−0.71**	**[−1.40, −0.01]**	**0.048**	**3.9%**	
PCoA axis 2	^NS^	^NS^	^NS^	^NS^	

## Discussion

In defining links between maternal ASB consumption and infant BMI, our results suggest that maternal consumption of ASB during pregnancy (1) may influence the establishment of the infant gut microbiome in infants diverging from what has previously been described as the typical microbiome maturation trajectory (Table 2, Figure 3a); and (2) is associated with an increase in infant BMI at one-year-old that may be mediated by succinate (Table 3). The estimated impact of maternal ASB consumption on the infant microbiome is notably smaller than other known drivers (which uniformly affected infants across all clusters) such as breastfeeding, birth mode, ethnicity, infant age, and intrapartum antibiotics (Figure 3a, eFigure 2). However, ASB consumption was also linked to differences in urine metabolites known to be produced by microbial metabolism of putrescine in the gut, supporting the role of ASBs in taxonomic and functional changes of the early life microbiome. To our knowledge, this is the first human study to report the association of maternal consumption of ASB, infant gut microbiome, and urine metabolites, and their potential influence on infant BMI. In light of recent data showing that ASB can drive dysregulation of energy metabolism in mice through changes in the gut microbiome,^24,27,30,31^ our study suggests that infants exposed to ASB through their mothers may be at higher risk of shifts in microbial community structure related to early-life predisposition to metabolic diseases.^32,33^

The first year of life has been suggested to be a “window of opportunity” for the training of the immune system through interactions between host cells, gut microorganisms, and microbial metabolites (see Arrieta *et al*.^34^ for a review). During this period, method of birth,^9,35^ infant nutrition,^16–18^ and antibiotic treatment^14^ are major drivers of infant gut microbiome establishment and trajectory, respectively, determining the initial set of pioneer species^36^ and stochastic perturbations potentially leading to dysbiosis (i.e. a state of community imbalance triggered by loss of taxa, diversity, and/or metabolic capacity).^37^ Our results confirm the significance of these factors in driving infant gut microbial community structure during the first year of life (Figure 3a). Most interestingly, although we included multiple variables describing antibiotic treatment either received by the mother of the infant, intrapartum antibiotics was identified as a strong determinant of microbial community structure. This effect was detected in clusters 1 and 2 only, suggesting its effect might resolve with time. In addition, intrapartum antibiotics could contribute to the altered microbial trajectory of cluster 2 and perhaps increase susceptibility to the effect of maternal ASB, which was only observed in this cluster.

In our study, broad shifts in bacterial community structure were significantly associated with infant BMI at one-year-old. We also identified nine bacterial taxa from *Bacteroides* sp. that were enriched (three ASVs) or depleted (six ASVs) at high levels of maternal ASB consumption, suggesting a mechanism of influence on infant weight gain involving specific taxa of the gut microbiome. The taxa *Akkermansia municiphila* and genus *Bacteroides* have previously been identified by various studies to be respectively decreased and enriched as a consequence of ASB consumption.^27,30,31,38^ Our results differ from previous findings for *A. municiphila* and suggest that *Bacteroides* patterns of enrichment or depletion might be species- or strain-specific, warranting further research with deeper resolution.

In contrast to the microbiome sequencing findings, functional links between ASB consumption and the gut microbiome were evident using the complete dataset. Untargeted metabolomic analysis yielded two related metabolites, spermidine and succinate, associated with ASB consumption in the urine of 3 M-old infants (Figure 4). Of these, succinate was significantly and positively associated with infant BMI at one-year-old (Table 3, Figure 4d). Both metabolites are derivatives of putrescine, a relevant polyamine exerting a wide array of biological functions (e.g. gene regulation, stress resistance, cell proliferation and differentiation).^39,40^ Spermidine is known to be produced by gut-colonizing bacteria and can have an impact on host metabolism (e.g. increasing glucose homeostasis and insulin sensitivity, reducing adiposity and hepatic fat accumulation) in obesity mouse models.^41,42^ Succinate is produced by bacterial fermentation of dietary fibers in the gut.^43^ High levels of succinate within the gut lumen have been related to dysbiosis, inflammatory bowel disease (IBD) and intestinal inflammation in animal models by activating immune cells via succinate receptor 1(SUCNR1).^43^ Of these two metabolites, succinate was also found to mediate 29% of the effect of ASB exposure on BMI at 1-year. Of interest, high level of circulating succinate has been previously linked to obesity in humans.^44^ This exciting finding suggests that a common gut microbial metabolite previously associated with human obesity may play a role in infant weight gain linked to ASB consumption. These novel findings support a functional role of the gut microbiome in mediating the impact of ASB exposure on infant weight.

As reported by Bian *et al*.^30,31^ in two studies with adult mice, and by Nettleton *et al*.^45^ in a study on dams and their offspring, ASB has been shown to alter gut bacterial community composition (increase of *Bacteroides* and reductions of *Lactobacillus* and *Clostridium*) and increase body weight in parallel with an enrichment of energy metabolism bacterial genes. The functional cluster analyses by Bian *et al*.^30,31^ revealed activation of genes related to carbohydrate absorption and increases in metabolic pathways related to glycolysis and sugar and xylose transport.^30^ Sucralose treatment resulted in an increase in bacterial pro-inflammatory mediator genes in mice.^31^ Likewise, Chi *et al*.^38^ found that consumption of the artificial sweetener neotame altered the alpha- and beta-diversity of mice gut microbiome, and led to a decrease in butyrate synthetic genes and changes to the fecal short chain fatty acids cluster.

Overall, accumulating evidence suggests that the alterations of host gut bacterial community structure through the consumption of ASB are reflected in bacterial and host metabolic gene clusters, which might explain the increase in weight gain. Based on this evidence and our current results, we hypothesize that gestational exposure to ASB impacts infant gut bacterial communities either indirectly through disruption of vertical transmission of the maternal microbiome, or directly through lactation during breastfeeding. Additional work will determine if the bacterial compositional and metabolic changes associated with high maternal ASB consumption in our study are causally implicated in energy metabolism dysregulation and infant body composition.

Overall, our study agrees with previous findings^3^ that maternal consumption of artificial sweeteners is associated with a higher BMI at one-year-old, and suggests that the infant gut microbiome could play a role in this effect, especially for susceptible infants displaying a disrupted maturation trajectory of their gut microbiome and a high relative abundance of *Bacteroides*. Our study confirms recent descriptions of infant microbiome development and confirms the influence of several known determinants of the gut microbiome during the first year of life^11–14,16,17,19^ including maternal antibiotics, breastfeeding, birth mode and ethnicity.

The major strength of our study is the combination of state-of-the-art community typing analysis of the gut bacterial communities combined with the standardized prospective evaluation of maternal ASB consumption. Limitations of our study lie in risk of measurement error in self-reported dietary exposures and our inability to distinguish between different types of ASB or account for artificial sweeteners in foods. Also, we did not assess maternal diet after delivery, so we could not directly investigate the impact of prenatal ASB exposure *in utero* versus postnatal exposure through lactation.^46,47^ In addition, we used 16S amplicon sequencing to characterize the gut bacterial communities. This method is limited in resolution as many recent studies have revealed that host-microbe and microbe-microbe interactions occur at species and subspecies-level variants.^48,49^ Finally, aside from the gut microbiome, various other physiological mechanisms are altered in rodent offspring after exposure to artificial sweeteners *in utero*^21–24^ (i.e. intestinal sugar absorption stimulation, increased postnatal weight gain, altered lipid profiles, downregulation of hepatic detoxification, and increased adulthood insulin resistance). Future work should explore if the infant gut microbiome may contribute to the physiologic effects of artificial sweeteners.

In this study, we characterized the infant gut microbiome composition and function of 100 infants and found evidence that maternal ASB consumption during pregnancy might have unforeseen effects on infant gut microbiome development and body mass index during the first year of life. As we face an unprecedented rise in childhood obesity and related metabolic diseases, further research is warranted to understand the impact of artificial sweeteners on gut microbiome and weight gain, especially during critical periods of early development.

## Material and methods

### Study design and population

We used data and samples collected through the CHILD Cohort Study,^50,51^ a Canadian general population birth cohort (3621 families recruited across four provinces) including singleton pregnancies (>35 weeks gestational age with no congenital abnormalities) enrolled from 2008 to 2012. From this cohort, we completed a case-control study by selecting 100 infants divided equally between mothers that reported little or no ASB consumption (less than one per month) or high ASB consumption (one or more per day) during pregnancy. The groups were balanced for six potential confounding factors known to influence the gut microbiome: infant sex, birth mode, breastfeeding at three and 12 months, maternal BMI, and antibiotic use in infants before 12 months (antibiotics before three months old was an exclusion criterion; Table 1). To characterize the gut microbiome, stool samples were acquired at three and 12 months of age for a total of 200 samples. This study was approved by the University of Calgary Conjoint Health Research Ethics Board (CHREB) and ethics committees at the Hospital for Sick Children, and the Universities of Manitoba, Alberta, and British Columbia. Written informed consent was obtained from mothers during enrollment to the CHILD Study.

### Maternal diet in pregnancy

Maternal dietary assessment in pregnancy has previously been described.^3^ Briefly, a food frequency questionnaire (FFQ) was completed during the second or third trimester and ASB consumption was evaluated using reports of “diet soft drinks or pop” (i.e. soda) (serving = 12 oz/one can) and “artificial sweetener added to tea or coffee” (serving = 1 packet). Other dietary variables included: sugar-sweetened beverages, Healthy Eating Index (HEI) total score (see eMethods) added sugar and total energy intake.

### Infant BMI

BMI was measured by CHILD staff to the nearest 0.1 kg around one year of age (mean = 12.0 months ± 0.8 [sd]) and height to the nearest 0.1 cm. Age- and sex-specific BMI-for-age z-scores were calculated following the World Health Organization reference.^52^

### Other variables

The following variables were considered in univariable analyses (see eMethods): (1) infant’s sex, age at sample collection, breastfeeding duration (BF duration; months), breastfeeding status at three months (BF at 3 M; yes or no), diet at three and six months (Diet at 3 M and Diet at 6 M; both defined in 8 categories allocated based on the presence in the infant’s diet of breastfeeding, formula, and solids), solids at three and six months (Solids at 3 M and Solids at 6 M), formula feeding at three months (FF at 3 M), number of antibiotic treatments received from six to twelve months (Child 6–12 abx), and secretor status (determined from the single nucleotide polymorphism rs601338 in the *FUT2* gene); (2) mother’s gestational diabetes, age, ethnicity, education, oral antibiotics received during gestation (Mother gestational abx), intrapartum antibiotics (Mother intrapartum abx), and secretor status (rs601338 SNP); (3) study site, presence of cats, dogs, and older siblings in the house.

### Fecal samples DNA extraction and sequencing

After collection, fecal samples were frozen and stored at −80ºC. We extracted gut microbial DNA from fecal samples using the DNeasy PowerSoil kit (QIAGEN) according to the manufacturer’s instructions and amplified the V4 region of the 16S rRNA gene to generate ready-to-pool dual-indexed amplicon libraries as described previously^53^ (see eMethods). Using the DADA2^54^ pipeline, the final dataset contained 4,553,000 quality sequences, a mean (range) of 6,509 (22,995–68,265) sequences per sample identified as 954 unique bacterial Amplicon Sequence Variants (ASVs). Samples contained a mean of 40 (10–95) unique ASVs per samples.

### Urine untargeted metabolomics

We have applied an untargeted quantitative metabolomics approach to analyze the samples using a combination of direct injection mass spectrometry with a reverse-phase LC-MS/MS custom assay. This custom assay, in combination with an ABSciex 4000 QTrap (Applied Biosystems/MDS Sciex) mass spectrometer, can be used for the identification and quantification of up to 150 different endogenous metabolites including amino acids, acylcarnitines, biogenic amines & derivatives, uremic toxins, glycerophospholipids, sphingolipids and sugars.^55,56^ The method combines the derivatization and extraction of analytes, and the selective mass-spectrometric detection using multiple reaction monitoring (MRM) pairs. Isotope-labeled internal standards and other internal standards are used for metabolite quantification. The custom assay contains a 96 deep-well plate with a filter plate attached with sealing tape, and reagents and solvents used to prepare the plate assay. First 14 wells were used for one blank, three zero samples, seven standards and three quality control samples. For all metabolites, samples were thawed on ice and were vortexed and centrifuged at 13,000x g. 10 µL of each sample was loaded onto the center of the filter on the upper 96-well plate and dried in a stream of nitrogen. Subsequently, phenyl-isothiocyanate was added for derivatization. After incubation, the filter spots were dried again using an evaporator. Extraction of the metabolites was then achieved by adding 300 µL of extraction solvent. The extracts were obtained by centrifugation into the lower 96-deep well plate, followed by a dilution step with MS running solvent. Mass spectrometric analysis was performed on an ABSciex 4000 Qtrap® tandem mass spectrometry instrument (Applied Biosystems/MDS Analytical Technologies, Foster City, CA) equipped with an Agilent 1260 series UHPLC system (Agilent Technologies, Palo Alto, CA). The samples were delivered to the mass spectrometer by an LC method followed by a direct injection (DI) method.

### Statistical analysis

We used Dirichlet Multinomial Mixtures (DMM) modeling^28^ on 16S rRNA gene sequencing data to identify clusters of similar bacterial community structure amongst our samples (a technique known as community typing analysis, increasingly used in human microbiome studies^10,57–59^). This technique is increasingly employed in microbiome studies for three reasons: (1) identification of unique microbial clusters is unsupervised; (2) cluster size depends on metacommunity variability; and (3) adequate explicit probabilistic model penalizes model complexity to optimize cluster number. The lowest Laplace approximation grouped our samples in four unique clusters (Figure 1–2 and eFigure 1).

The distribution of variables as well as the variation in bacterial richness (Chao 1), alpha-diversity (Shannon index), and community evenness (Shannon index/log_n_(species richness)) across the DMM clusters were examined by non-parametric Kruskal-Wallis tests followed by post-hoc Dunn tests or generalized linear models (glm) with a binomial/logistic distribution. To explore the changes in taxonomical community structure at a fine scale, we tested for significant differences in the relative abundance of the 10 most dominant bacterial genera across clusters using non-parametric Kruskal-Wallis tests followed by post-hoc Dunn tests with Benjamin-Holmes False Discovery Rate (FDR) correction. To account for potential heteroskedasticity in bacterial community dispersion between groups and avoid the loss of information through rarefaction,^60^ we performed a variance stabilizing transformation^60,61^ prior to any statistical tests on beta-diversity. To select variables that could be drivers of infant gut bacterial community structure, we tested for correlations between our variables and community scores on the Principal Component Analysis (PCoA) ordination axes in univariable models (*envfit* function of vegan^62^). The relative influence of the significant drivers of gut bacterial community structure was then assessed statistically in multivariate models using a Permutational Multivariate Analysis Of Variance (PERMANOVA based on Bray-Curtis dissimilarities; *adonis* function of vegan^62^) with 999 permutations and visualized using PCoAs. We used DESeq2 (with Benjamini-Hochberg False Discovery Rate (FDR) correction) to test for differentially abundant bacterial taxa according to maternal ASB consumption on the 100 most relatively abundant bacterial taxa to limit spurious significance driven by very rare ASVs. Finally, we used linear models on the 3 M and 12 M-old samples to test for the influence of maternal ASB consumption, urine metabolites, and microbial ordination axes (PCoA1 and PCoA2) on infant BMI z-score. We performed the mediation analysis using “*Succinate*” as mediator and “*ASB*” as mediated with the *mediation* package. All analyses and graphs were computed in R version 3.6.1 (R Development Core Team; http://www.R-project.org) and MetaboAnalyst^29^ for the metabolomics.

## Supplementary Material

Supplemental Material

## Data Availability

Raw sequences have been deposited on NCBI public repository (Bioproject #PRJNA624780). The R code, metadata, community matrix and taxa matrix are available on github https://github.com/isabelle-laforest/CHILD_GutMicrobes.
