# Supplementary material for: Maternal consumption of artificially sweetened beverages during pregnancy is associated with infant gut microbiota and metabolic modifications and increased infant body mass index
Source: Gut Microbes. 2020 Dec 31;13(1):1857513. doi: 10.1080/19490976.2020.1857513 (PMC7781635; doi:10.1080/19490976.2020.1857513)
Supplement: Supplemental Material [file KGMI_A_1857513_SM0710.pdf]

ONLINE-ONLY SUPPLEMENTS

eMethods

eTable 1. Summary statistics of the age at sampling per cluster. No statistical differences were detected (non-parametric Kruskal-Wallis test; 3 months: P=0.47; 12 months: P=0.53).

eFigure 1. Community typing analysis identified four unique clusters based on genus level bacterial community composition. Dirichlet Multinomial Mixtures (DMM) modelling of 16S rRNA gene sequencing data (n = 198) of 100 infants with two samples each. The entire dataset formed four distinct clusters based on lowest Laplace approximation. Heat map showing the relative abundance of the 15 most dominant bacterial genera per DMM cluster.

eFigure 2. Drivers of Infant Gut Bacterial Community Structure Differ Between Clusters. Univariate models showing significance and explained variance of 31 variables on bacterial community structure across all data and each cluster subset. Horizontal bars show the amount of variance (R^2) explained by each covariate in the model as determined by *envfit*. Asterisk denotes the significant covariates in each data subset (P<0.05).

eFigure 3. Microbial functional profiles (metabolomics) differ across clusters from 3- (cluster 1) to 12-months old (cluster 4). (A) Heatmap of the top 25 metabolites from infant urine samples. (B) Box plots significant statistical differences in 20 metabolites between DMM cluster. The central line denotes the median, the boxes cover the 25th and 75th percentiles, and the whiskers extend to the most extreme data point, which is no more than 1.5 times the length of the box away from the box. Points outside the whiskers represent outlier samples. Letters denoted significant differences (one-way ANOVA test followed by post-hoc test of Fisher's LSD with FDR correction (Benjamini-Hochberg method; P<0.05).

eFigure 3. The main changes in bacterial community structure are defined by infant age. PCoA of the variation in beta-diversity of infant gut bacterial communities (Bray-Curtis dissimilarities) among samples from the complete dataset. Points represent samples and are colored by infant age (blue for three-month-old and yellow for twelve-month-old) and arrows represent the significant (p<0.01) correlations between PCoA axes versus the relative abundances of bacterial classes in communities.

eREFERENCES

eMETHODS

Covariates

Hospital records provided information on maternal diabetes, infant sex, and maternal age. Maternal BMI was calculated as reported in Azad *et al.*[1] from measured height and self-reported pre-pregnancy weight (n = 66) or estimated from measured weight at 1 year after birth if mothers could not recall their pre-pregnancy weight (n = 34). A questionnaire filled during pregnancy collected information on location (site), maternal ethnicity (Asian, Caucasian, First Nations, other), older siblings, and maternal education. Breastfeeding at three months (BF at 3M), formula feeding at three months (FF at 3M), diet at three months (Diet at 3M) and six months (Diet at 6M), and timing of the introduction of solid foods (Solids at 3M and Solids at 6M) were reported by a standardized questionnaire at three, six, and 12 months after birth. Duration of breastfeeding (BF duration) is a continuous variable representing the age in months at breastfeeding cessation, or "12 months" if still breastfeeding after 1 year. The Healthy Eating Index (HEI) measures diet quality using a scoring system from 0 to 100. A perfect overall HEI score of 100 reflects alignment with key dietary recommendations from the *Dietary Guidelines for Americans*[2]. The HEI2010 score is the sum of 12 components including total fruits, total vegetables, whole grains, dairy, proteins, fatty acids, etc. Missing values were imputed by multiple imputation (n = 48 values across 33 covariates) over 20 imputed data sets with fully conditional specification (chained equations) using all covariates.

Sequence analysis

Amplicons were quantified with PicoGreen (Invitrogen) and diluted to 20 ng/µl for sequencing. Microbial sequencing was performed on the MiSeq Illumina platform at the Centre for Health Genomics and Informatics Facility, at University of Calgary. The pooled and indexed library set was denatured, diluted, and sequenced in paired-end modus on an Illumina MiSeq (Illumina Inc., San Diego, USA). Sequences were checked for quality, trimmed, merged, and checked for chimeras using the DADA2[3] and phyloseq[4] packages for R (R Development Core Team; http://www.R-project.org). We built a bacterial community matrix from the resulting unique set of Amplicon Sequence Variants (ASVs) identified. After excluding two samples based on sequencing read pairing and quality, we analyzed the infant gut bacterial communities of 198 stool samples from 100 infants. To reduce biases introduced by DNA amplification (i.e. PCR) and by sequencing errors, we excluded any ASV that was found less than 20 times in the whole dataset (across all samples).

Cluster community composition

In Cluster 1 (3M infants), bacterial taxonomic composition showed a significant higher relative abundance of genera *Escherichia* and *Bifidobacterium*, as well as a lower relative abundance of the genera *Bacteroides*, *Ruminococcus*, *Akkermansia*, *Faecalibacterium*, and *Parabacteroidetes* (Figure 2). This cluster also had the lowest species richness and alpha-diversity (Figure 1IJ), consistent with previous studies of the early-life gut microbiome[5]. In cluster 2 (mixed 3M-12M), bacterial taxonomic composition was dominated by the genus *Bacteroides* (Figure 2-3A). Cluster 3 (mixed 3M-12M, with the highest proportions of Cesarean deliveries, formula feeding, and intrapartum antibiotics) displayed a higher relative abundance of *Escherichia*, *Bifidobacterium*, *Ruminococcus*, *Clostridium*, and *Veillonella*, while showing a lower relative abundance of *Bacteroides* (Figure 2). This cluster displayed a high alpha-diversity (Figure 1I) and higher evenness than clusters 1-2 ($P < 0.001$). Finally, bacterial taxonomical composition of cluster 4 (12M infants) was dominated by the genus *Bacteroides* but also by a higher relative abundance of *Faecalibacterium* (Figure 2). Cluster 4 displayed the highest alpha-diversity and species richness (Figure 1IJ), as well as a higher evenness than clusters 1-2 ($P < 0.001$). Across all samples, the most ubiquitous ASV was identified as *Akkermansia municiphila* (present in 154/198 samples).

eTable 1. Summary statistics of the age at sampling per cluster. No statistical differences were detected (non-parametric Kruskal-Wallis test; 3 months: P=0.47; 12 months: P=0.53).

Cluster	Age at 3 months sample		Age at 12 months sample	
	Mean	Sd	Mean	Sd
1	3.34	0.54	12.16	0.95
2	3.30	0.61	12.03	0.84
3	3.23	0.59	11.91	0.73
4	3.25	0.62	12.22	0.97

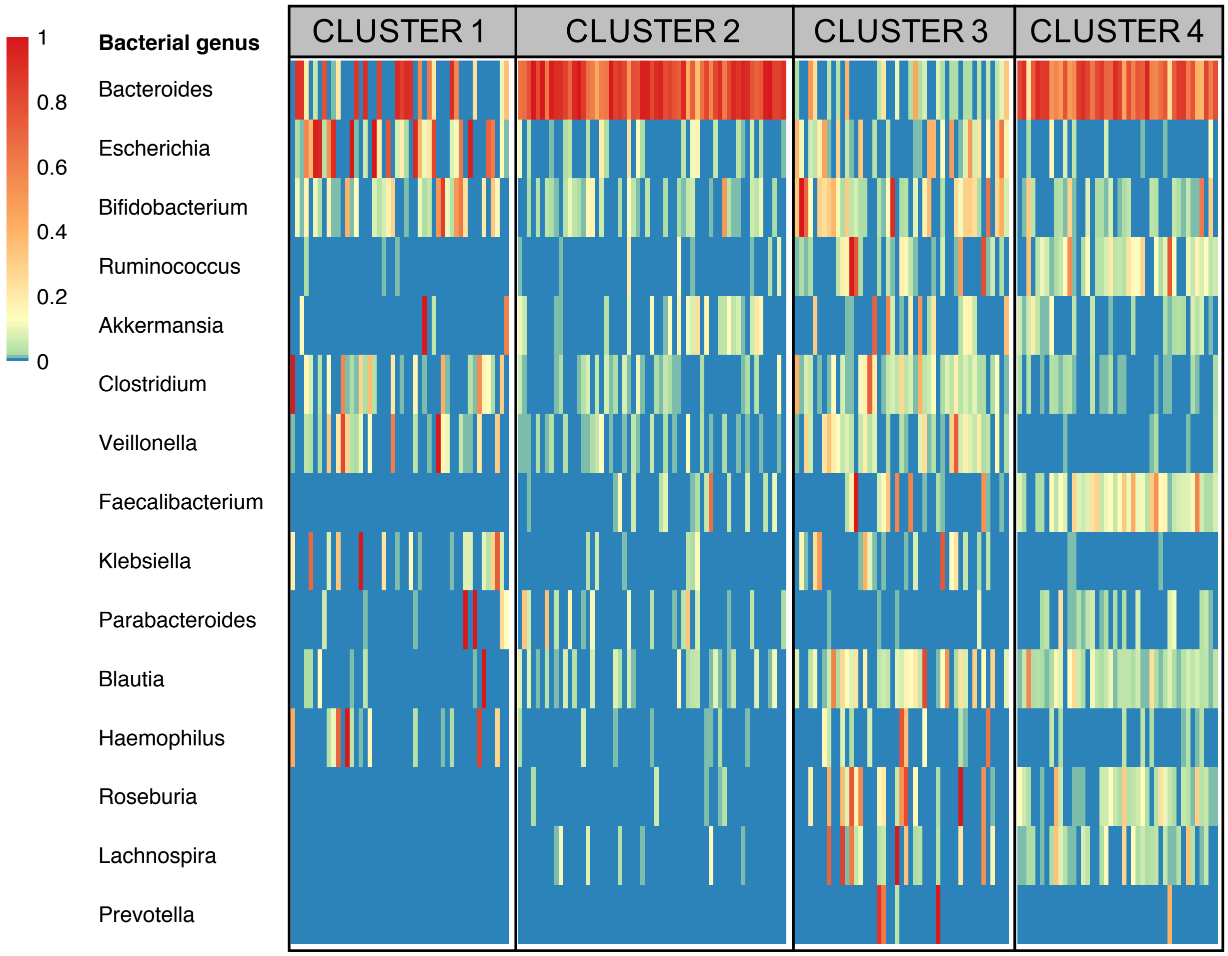

eFigure 1. Community typing analysis identified four unique clusters based on genus level bacterial community composition. Dirichlet Multinomial Mixtures (DMM) modelling of 16S rRNA gene sequencing data (n = 198) of 100 infants with two samples each. The entire dataset formed four distinct clusters based on lowest Laplace approximation. Heat map showing the relative abundance of the 15 most dominant bacterial genera per DMM cluster.

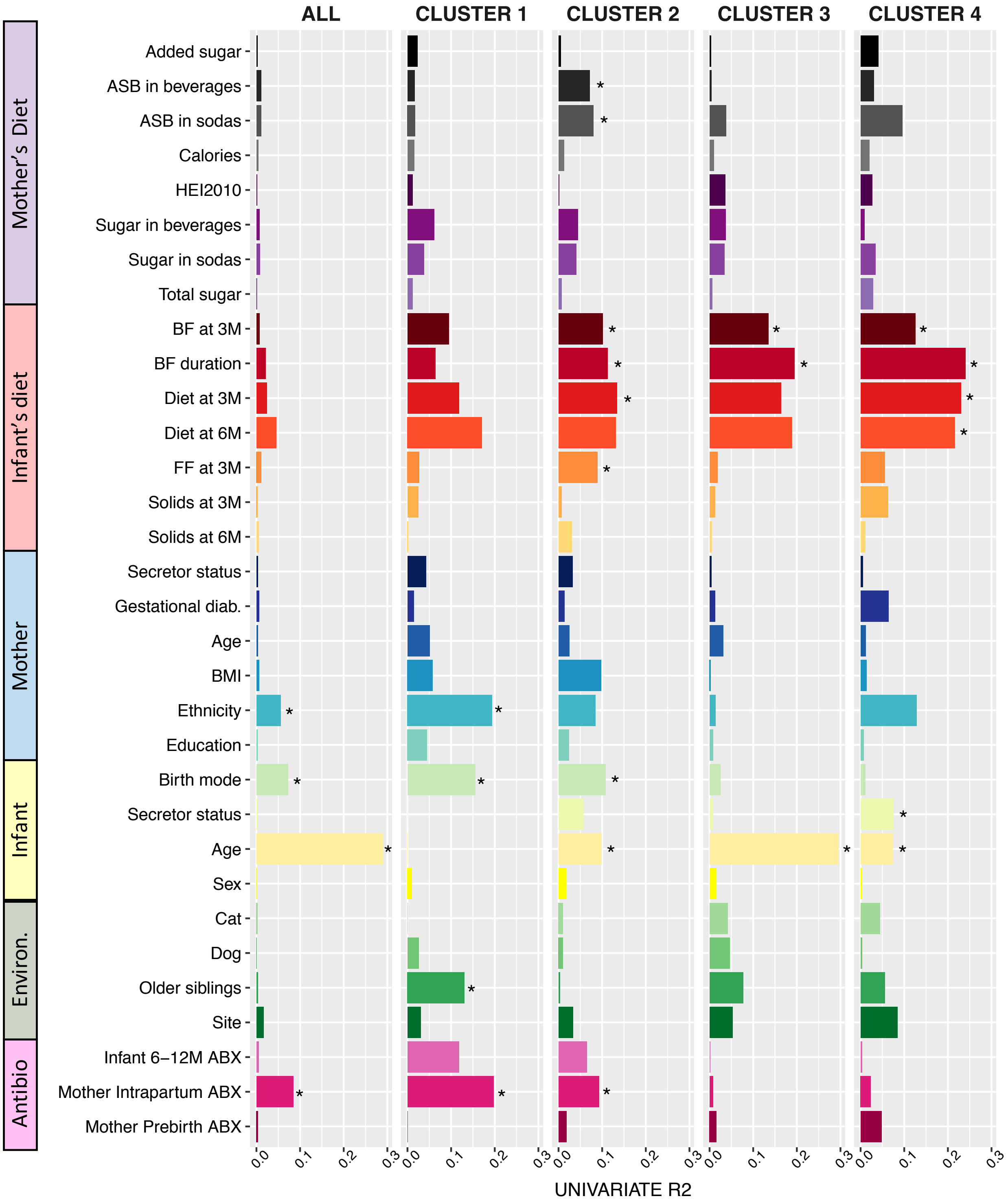

eFigure 2. Drivers of Infant Gut Bacterial Community Structure Differ Between Clusters. Univariate models showing significance and explained variance of 31 variables on bacterial community structure across all data and each cluster subset. Horizontal bars show the amount of variance (R^2) explained by each covariate in the model as determined by *envfit*. Asterisk denotes the significant covariates in each data subset (P<0.05).

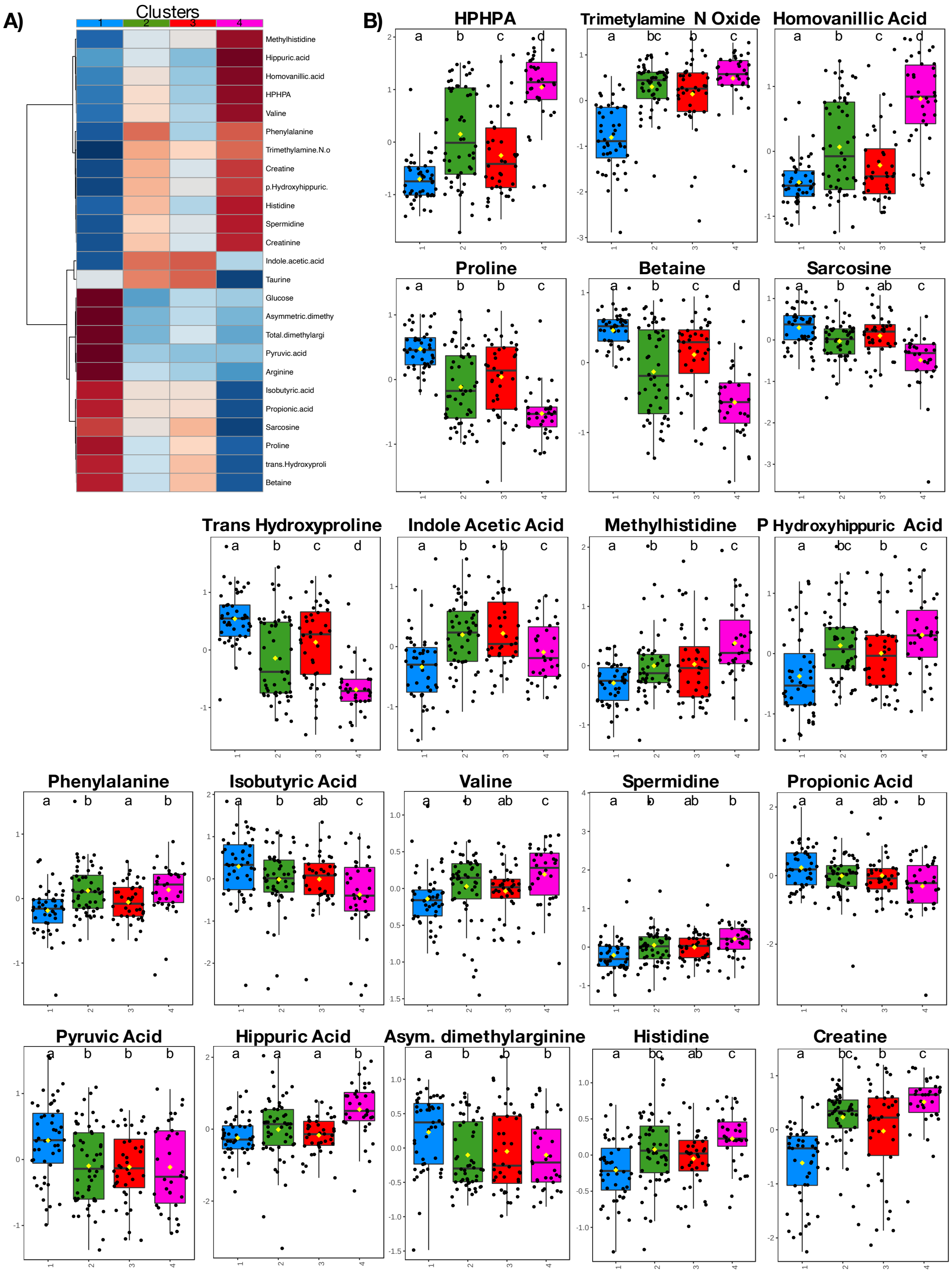

eFigure 3. Various metabolomic profiles across clusters from 3- (cluster 1) to 12-months old (cluster 4). (A) Heatmap of the top 25 metabolites from infant urine samples. (B) Box plots significant statistical differences in 20 metabolites between DMM cluster. The central line denotes the median, the boxes cover the 25th and 75th percentiles, and the whiskers extend to the most extreme data point, which is no more than 1.5 times the length of the box away from the box. Points outside the whiskers represent outlier samples. Letters denoted significant differences (one-way ANOVA test followed by post-hoc test of Fisher's LSD with FDR correction (Benjamini-Hochberg method; P<0.05).

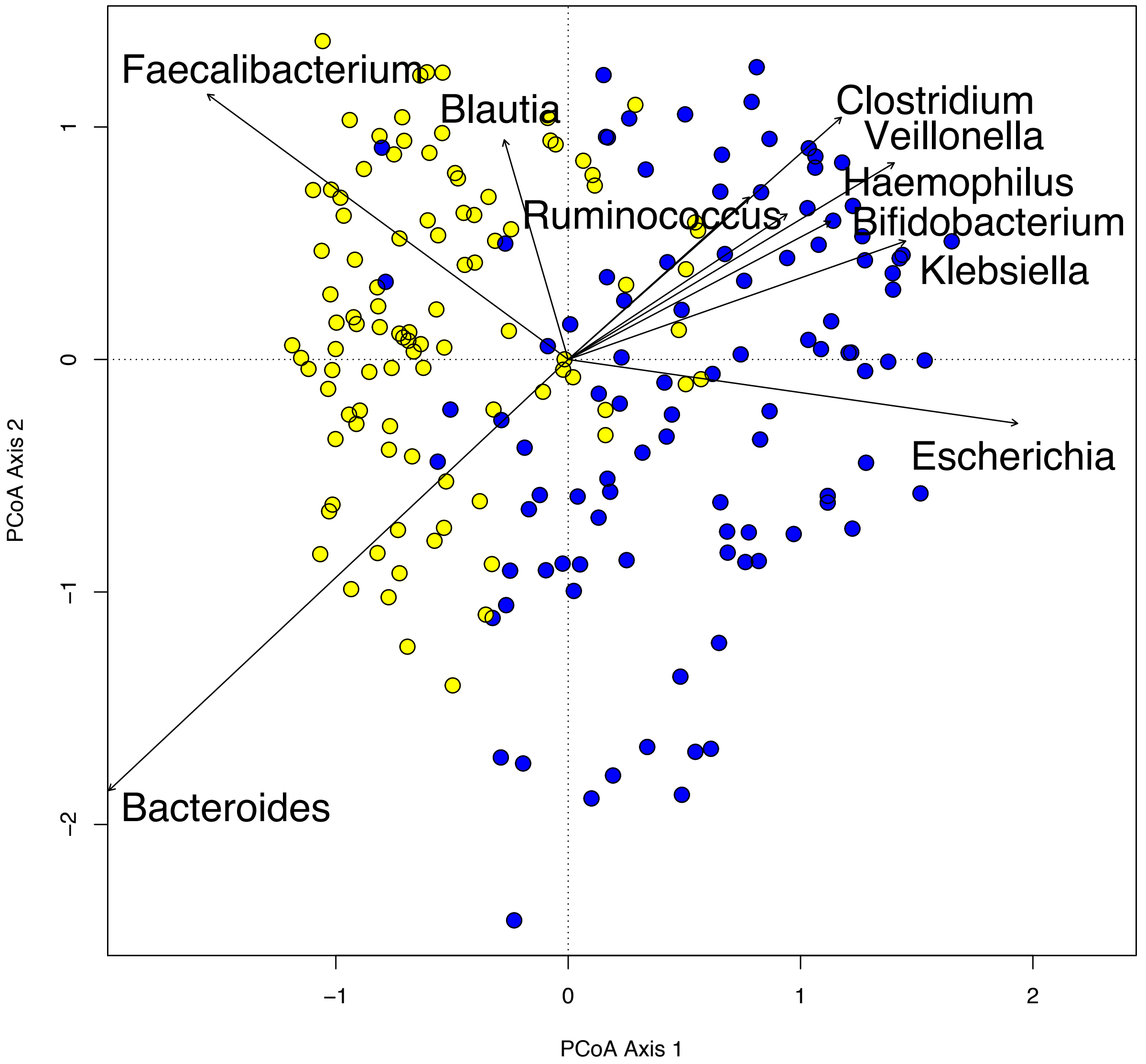

eFigure 4. The main changes in bacterial community structure are defined by infant age. PCoA of the variation in beta-diversity of infant gut bacterial communities (Bray-Curtis dissimilarities) among samples from the complete dataset. Points represent samples and are colored by infant age (blue for three-month-old and yellow for twelve-month-old) and arrows represent the significant ($p<0.01$) correlations between PCoA axes versus the relative abundances of bacterial classes in communities.

eREFERENCES

1. Azad MB, Sharma AK, de Souza RJ, et al. Association Between Artificially Sweetened Beverage Consumption During Pregnancy and Infant Body Mass Index. *JAMA Pediatr.* 2016;170(7):662-670.
2. U.S. USDoAaUSDoHaHS. *Dietary Guidelines for Americans.* 7th Edition ed. Washington, DC: U.S. Government Printing Office2010.
3. Callahan BJ, McMurdie PJ, Rosen MJ, Han AW, Johnson AJ, Holmes SP. DADA2: High-resolution sample inference from Illumina amplicon data. *Nat Methods.* 2016;13(7):581-583.
4. McMurdie PJ, Holmes S. phyloseq: an R package for reproducible interactive analysis and graphics of microbiome census data. *PLoS One.* 2013;8(4):e61217.
5. Laforest-Lapointe I, Arrieta MC. Patterns of Early-Life Gut Microbial Colonization during Human Immune Development: An Ecological Perspective. *Front Immunol.* 2017;8:788.
